# Effectiveness and infectious complications of BCMA T‐cell engagers in treating multiple myeloma: Real‐world evidence from Sweden

**DOI:** 10.1002/cam4.7048

**Published:** 2024-04-23

**Authors:** Katarina Uttervall, Love Tätting, Konstantinos Lemonakis, Mousa Majd, Jacob Crafoord, Mikael Olsson, Ulf‐Henrik Mellqvist, Markus Hansson, Hareth Nahi

**Affiliations:** ^1^ Center for Hematology and Regenerative Medicine, Department of Medicine, Huddinge Karolinska Institutet Stockholm Sweden; ^2^ Department of Haematology in Linköping, and Department of Biomedical and Clinical Sciences Linköping University Linköping Sweden; ^3^ Department of Hematology Lund University Lund Sweden; ^4^ Department of Medicine Orebro University Hospital Orebro Sweden; ^5^ Section of Hematology Department of Internal Medicine Hallands Sjukhus Varberg Varberg Sweden; ^6^ Section of Hematology and Coagulation, Department of Medicine Sahlgrenska University Hospital Gothenburg Sweden

**Keywords:** bispecific antibodies, immunotherapy, multiple myeloma, real‐world data

## Abstract

**Background:**

Multiple myeloma (MM), an incurable disease characterized by frequent relapses and a need for multiple treatments, often progresses to a relapse/refractory status resistant to all available drugs and drug classes. Bispecific antibodies, specifically BCMA T‐cell engagers, have emerged as effective treatments for MM, demonstrating impressive efficacy. However, these treatments can adversely affect the immune system, increasing vulnerability to infections.

**Methods/Results:**

This study evaluated the efficacy and safety of BCMA T‐cell engagers in 58 Swedish patients with poor MM prognosis. The patients exhibited a 69% overall response rate, with 69% survival and 60% progression‐free survival at 15 months.

**Conclusions:**

Despite the risk of infectious complications, the prognosis of MM patients can be significantly improved with vigilant monitoring and proactive management of infections. This real‐world data highlight the potential of BCMA T‐cell engagers in treating MM, emphasizing the need for careful patient monitoring to mitigate infection risks.

## 1 INTRODUCTION

Multiple myeloma (MM) is a hematological malignancy characterized by the proliferation of malignant plasma cells in the bone marrow, leading to various clinical manifestations and complications. Despite advances in treatment, MM remains incurable, with many patients relapsing or becoming refractory to existing therapies.^1^ With the recent introduction of anti‐CD38 monoclonal antibodies (mAbs), daratumumab, immunotherapy has rapidly become indispensable in the management of the disease. Anti‐CD38 antibody is, according to different guidelines, recommended in the front treatment of all MM patients, both high dose eligible and non‐eligible patients, together with a proteasome inhibitor (PI) and an immunomodulatory drug (IMiD). This marked a significant advancement in MM treatment, improving patient outcomes considerably. However, the emergence of resistance to these therapies has been a growing concern, highlighting the need for new treatment strategies.^2^ In relapsed/refractory MM (RRMM), there is no standard of care. Recommendations in the national and international guidelines are based on earlier treatment/s and the outcome of these drug combinations based on IMiDs, PIs, anti‐CD38 mABs, HDAC inhibitors, chemotherapy, and corticosteroids. Other emerging treatment options include CAR‐T and bispecific antibodies (BsAbs) and for patients harboring t(11;14) BCL‐2 inhibitors^3^ venetoclax, alone or in combination is sometimes recommended. Despite all available treatments for MM, most MM patients relapse and become refractory; thus, there is an unmet need for new medications with a different mode of action.

Bispecific antibodies, especially those targeting the B‐cell maturation antigen (BCMA), have recently emerged as a promising class of therapeutics in MM. These agents, by engaging both cytotoxic T cells and myeloma cells, offer a targeted approach to therapy. Teclistamab and elranatamab, two such BsAbs, have demonstrated considerable efficacy in recent clinical trials.^4^, ^5^ However, the clinical use of BsAbs is not without challenges, particularly regarding their safety profile. Experience with BCMAxCD3 BsAbs shows a quick decline in IgA, IgM, IgG, and free light chains in a majority of patients, putting them in a deep hypogammaglobulinemic state.

This study reports on the real‐world experience on efficacy, safety, and effect of introducing up‐front immunoglobulin substitution. By analyzing real‐world data from patients treated with BsAbs in Sweden within compassionate‐use programs, we seek to provide insights into optimizing the therapeutic use of these novel agents in clinical practice.

## 2 MATERIALS AND METHODS

### 2.1 Patients

This study included 58 patients treated with BsAbs targeting BCMA, specifically teclistamab or elranatamab, as part of compassionate‐use programs in Sweden. These patients were treated in nine hospitals across the country, encompassing both university and regional medical centers, between June 2021 and June 2023. Eligibility for inclusion followed disease criteria established in previously described phase II studies.^4^, ^5^

### 2.2 Treatment

Initially, all patients were hospitalized for the step‐up phase to closely monitor and manage immediate adverse reactions. Treatment protocols for teclistamab and elranatamab followed those outlined in the respective phase II trials.^4^, ^5^ These included, as earlier reported, specific dosing schedules and adjustments based on patient tolerability. After the step‐up phase, patients continued their treatment on an outpatient basis, with regular follow‐ups to monitor response and manage any adverse effects.

### 2.3 Study design and overview

The study was approved by the Swedish Ethics Committee (Dnr 2023‐03123‐01). It adhered to the principles of the Declaration of Helsinki, the International Conference on Harmonization, and the Guidelines for Good Clinical Practice. Written informed consent was obtained from all participants. This cohort study included patients with exhausted treatment options who were part of a compassionate‐use program offered by Janssen Biotech (Solna, Sweden) and Pfizer (Solna, Sweden).

### 2.4 Statistical analyses

Descriptive statistics was done in Microsoft Excel. Time‐to‐event analysis was done in IBM SPSS with Kaplan–Meier analysis. Plots were done in IBM SPSS (version 29.0.1.0 (171)), Graphpad Prism and R (Version 4.3.1, R Statistical Foundation).

## 3 RESULTS

### 3.1 Patient demographics and baseline characteristics

The study cohort comprised 58 patients with a median age of 62 years (range 37–78 years). The majority of the patients had a performance status of ECOG 2 or lower, with a small proportion (5%) at ECOG 3. All patients had measurable disease per IMWG guidelines. Prior treatments included a range of therapies such as IMiDs, PIs, CD38 mABs, and autologous as well as allogeneic stem cell transplantation. Patient characteristics are summarized in Table 1.

**TABLE 1:** Baseline characteristics and prior treatment.

Characteristics	Total (*n* = 58)
Median age (range), years	62.9 (37–80)
Male, *n* (%)	35 (60)
ECOG performance status, *n* (%)	
0	13 (22.4)
1	30 (52)
2	10 (17.2)
3	3 (5)
Unknown	2 (3.4)
Type of myeloma, *n* (%)	
IgG	26 (45)
IgA	14 (24)
IgD	1 (2)
Light chain	15 (26)
Unknown	2 (3)
ISS disease stage, *n* (%)	
I	20 (34)
II	11 (19)
III	15 (26)
Unknown	12 (21)
Cytogenetic risk, *n* (%)	
Standard	22 (38)
High^a^	15 (26)
Missing	21 (36)
Median no. of prior antimyeloma lines of therapy (range)	7 (3–17)
Prior stem cell transplant, *n* (%)	45 (78)
Exposure status, *n* (%)	
Triple class^b^	58 (100)
Penta‐drug^c^	54 (93)
Refractory status, *n* (%)	
Triple class	52 (90)
Penta‐drug	41 (71)

^a^ Includes: add(1q), t(4;14), t(14;16) and del(17p) chromosomal abnormalities.

^b^ Triple class refers to at least one proteasome inhibitor, one immunomodulatory drug and one anti‐CD38 antibody.

^c^ Penta‐drug refers to at least two proteasome inhibitors, two immunomodulatory drugs and an anti‐CD38 antibody.

### 3.2 Response to treatment

Of the 58 patients treated with BsAbs, 40 (69%) demonstrated a response according to the International Myeloma Working Group (IMWG) guidelines. Specifically, 28 patients (48%) achieved biochemical complete remission (CR), 4 (7%) very good partial remissions (VGPR), and 8 (14%) partial remissions (PR). The remaining patients showed minimal response (MR), stable disease (SD), or progressive disease (PD).

### 3.3 Survival outcomes

**FIGURE 1:**
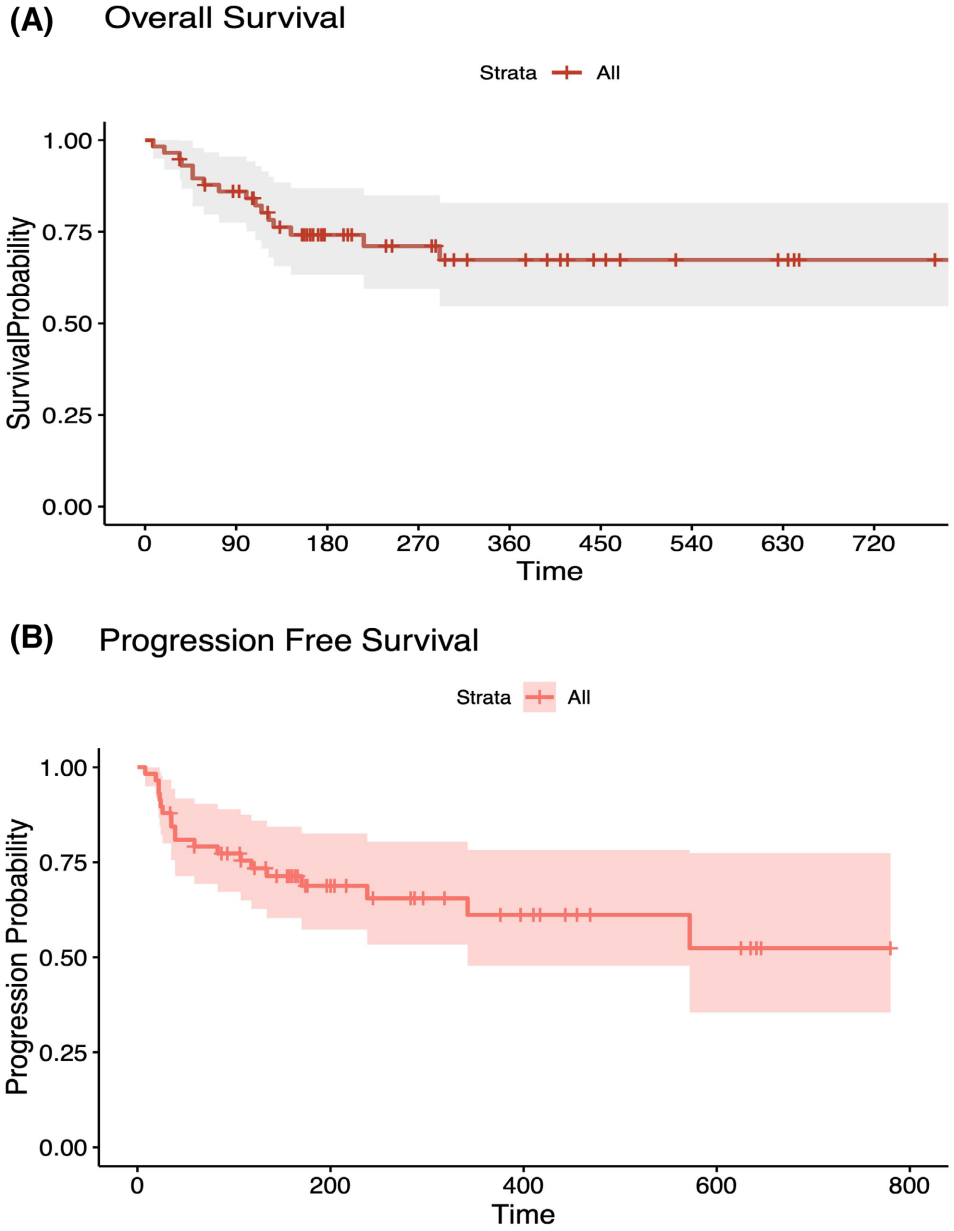
(A); Overall survival and (B); progression‐free survival since the start of treatment with the bispecific antibody.

The median follow‐up time for the entire cohort was 15.1 months. As of the data cutoff, 17 patients (29%) had deceased. Thirty‐two patients (55%) are still undergoing treatment. Among the 26 patients (45%) who discontinued treatment, 20 did so due to disease progression, and 6 because of adverse events (AE). At 15 months, the median overall survival (OS) rate was 69%, and the median progression‐free survival (PFS) rate was 60% (Figure 1).

### 3.4 Safety

The treatment‐emergent adverse events (TEAEs) predominantly included cytopenias and cytokine release syndrome (CRS). Hematologic TEAEs such as neutropenia, anemia, and thrombocytopenia were common. CRS was observed in 62% of patients, primarily grade 1, with only one patient developing grade 2 CRS (Table 2). The median time to CRS onset was 4 h post‐BsAb administration, with a median duration of 6 h. CRS incidents were mostly reported after the first and second priming doses of BsAb. In total, 52 CRS incidents were reported in 36 patients: 34 incidents (65%) occurred after the first priming dose, 16 (31%) after the second priming dose, one (2%) after the first full dose, and one (2%) after the second full dose. Additionally, immune effector cell‐associated neurotoxicity syndrome (ICANS) grade 1 was reported in one patient. The patient that experienced ICANS was also the one to experience the only observed event of CRS grade 2.

The treatment of CRS was according to the treating physician's preferences and consisted of tocilizumab in five patients (14%), dexamethasone (8–12 mg) in 24 (67%) and only supportive care (paracetamol) in 7 (19%).

**TABLE 2:** Treatment‐emergent adverse events.

	Any grade, *n* (%)	Grade 3 or 4, *n* (%)
Any treatment‐emergent adverse event	58 (100)	40 (71.7)
Hematologic		
Anemia	25 (43)	10 (17)
Neutropenia	30 (52)	25 (43)
Thrombocytopenia	21 (36)	9 (16)
Nonhematologic		
Cytokine release syndrome	36 (62)	0
ICANS	1 (2)	0
Fatigue	5 (9)	0
Dermatitis	4 (7)	0
Pyrexia	3 (5)	0
Neuropathy	4 (7)	0
Renal failure	2 (2)	0
Pulmonary embolism	1 (2)	0
Diarrhea	1 (2)	0
COVID‐19 related	11 (19)	1 (2)

### 3.5 Infections

**FIGURE 2:**
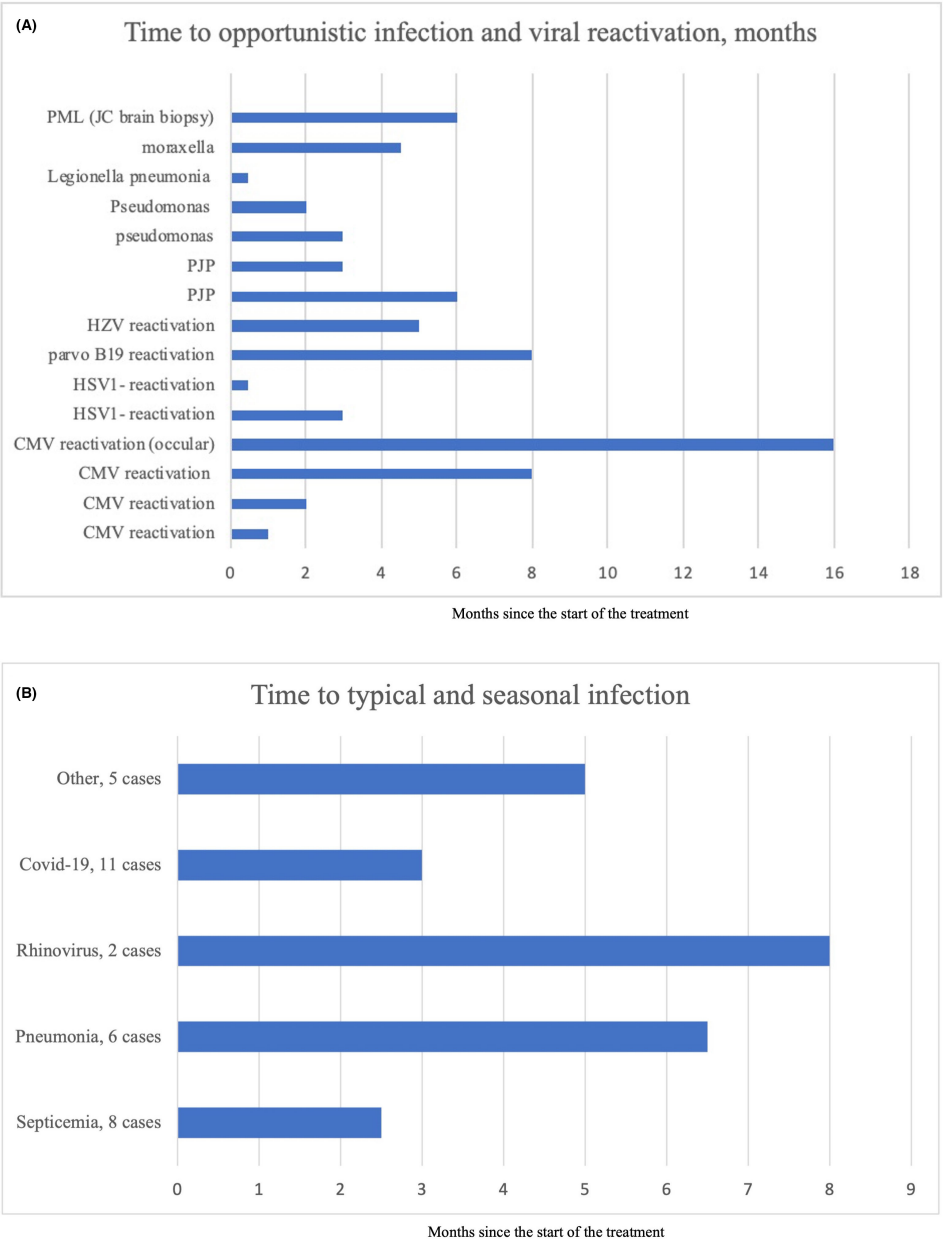
Time elapsed from the start of the treatment to infection. (A); viral reactivation and opportunistic infection and (B); typical and seasonal infections.

A total of 57 infections were reported in 28 patients (53%), including pneumonia, upper respiratory tract infection, urinary tract infection, septicemia, and COVID‐19. Virus reactivations such as cytomegalovirus (CMV), herpes simplex virus 1, herpes zoster virus, and parvovirus B19 were also noted. Opportunistic infections included pneumocystis jiroveci pneumonia (PJP), pseudomonas, legionella, moraxella, and progressive multifocal leukoencephalopathy (PML), which was diagnosed after a brain biopsy revealed JC virus. This patient had previously undergone allogeneic transplantation, discontinued treatment and subsequently passed away. Two cases of PJP were reported; neither patient had started prophylaxis before the infection (Figure 2).

Six patients were withdrawn from treatment, and two deaths were attributed to infections. Two patients suffered from COVID‐19: one had disease progression before contracting COVID‐19 and was in palliative care, while the other was alive at the data cutoff but ended treatment due to prolonged COVID‐19 infection. One patient, in a very good partial response (VGPR) but with grade 3 neutropenia, died from septicemia. Two patients with CMV reactivation discontinued treatment but were still alive at the data cutoff.

In Sweden, immunoglobulins were not routinely administered early but then incorporated as standard prophylaxis among all patients receiving BsAb as infection risk was recognized. Twenty‐two patients were treated between June 2021 and June 2022, 15 of them suffered from infections (68%), and 15 of the 36 patients (42%) treated after June 2022 suffered from infections.

The infections prophylaxis consisted of seasonal vaccination of influenza and COVID‐19, valaciclovir, trimethoprim/sulfamethoxazole, immunoglobulins (monthly) and in few patients even oral antibiotics were added (especially in cases with neutropenia). To start with these measures were adapted during the treatment but later on the prophylaxis was started as soon as the treatment with BsAb was initiated.

## 4 DISCUSSION

Multiple myeloma remains an incurable disease, with the majority of patients experiencing numerous relapses and requiring various treatments over time. Many patients eventually reach a state where they are deemed relapsed/refractory to all available drug classes. Currently, there is no standard of care for patients who are triple class refractory to multiple myeloma treatments, and even fewer options exist for those who are penta‐drug refractory. Both the rate and duration of response significantly decrease in these cases, with PFS often becoming less than 1 year.^1^, ^6^ Bispecific antibodies, which are in late‐stage clinical development, have demonstrated high efficacy in the treatment of RRMM. Phase II trials, MajesTEC‐1 for teclistamab and MagnetisMM‐3 for elranatamab,^4^, ^5^ which were instrumental in obtaining FDA approval, showed overall response rates of 63% and 61%, respectively. Furthermore, the median duration of PFS was reported to be 11.3 months for teclistamab and 50.9% (median not reached) at 15 months for elranatamab.^4^, ^5^

This study corroborates the previously mentioned high response rates in a real‐world setting, particularly among heavily pretreated patients. In this analysis, 90% of the patients had triple class refractory and 71% had penta‐drug refractory RRMM. Additionally, 22% of the patients had an ECOG status of 2 or more, 28% were classified as International Staging System disease stage III, and 26% exhibited high‐risk cytogenetic markers at diagnosis. Despite these unfavorable prognostic indicators, 40 patients (69%) responded to treatment, achieving at least a partial response (PR), with 55% experiencing a very good partial response (VGPR) or better. The OS and PFS rates were 69% and 60%, respectively, at 15 months.

The most common TEAEs in our cohort were CRS, hematologic‐related events, and infections. Adhering to trial protocol measures to mitigate CRS, such as premedication with corticosteroids, paracetamol, and antihistamines, along with a two‐step‐up priming dose regimen of both teclistamab and elranatamab during the first week of treatment, resulted in a CRS incidence of 62%. All but one incident was of grade 1 severity. Two patients experienced CRS beyond the second priming dose, implying that an outpatient schedule of priming is feasible. Immune effector cell‐associated neurotoxicity syndrome (ICANS) was rare, occurring in only one patient (2%) and was limited in severity to grade 1. While both study protocols initially required hospitalization for the step‐up priming doses, 65% and 31% of CRS events in our cohort occurred after the first and second priming doses, respectively. The median time to CRS onset was 24 h, with a median resolution time of 6 h, significantly shorter than early reports suggest.^4^, ^5^ The predictable and manageable nature of CRS and ICANS underscores the potential for outpatient administration and highlights the need for clinical trials to explore this further.

Previous treatments with IMiDs and PIs in RRMM have led to an increased incidence of infections compared to conventional therapies.^7^ Moreover, with the widespread use of mAb treatment, a high rate of infection and viral reactivation was observed.^8^ The fourfold increase in herpes zoster reactivation associated with bortezomib treatment is well‐documented.^2^ Additionally, CMV reactivation in hematological malignancies, especially in MM patients undergoing treatment, is a recognized complication.^9^ In our cohort, we observed high rates of infections, ranging from seasonal viruses and infections typical in myeloma such as pneumonia, as well as viral reactivations and opportunistic infections. One patient experienced PML due to JC viral infection, a rare but known issue in immunocompromised individuals. Two patients developed PJP before the initiation of prophylaxis.

Bispecific antibody treatments are known to cause hypo‐/agammaglobulinemia, B/T‐cell depletion, and neutropenia. Despite adequate vaccination and preemptive therapy for infections, patients still develop infections and viral reactivations, potentially increasing mortality rates. Strategies to bolster the patient's immune system, such as careful monitoring for CMV and pausing BsAb treatment until recovery, earlier initiation of viral and bacterial prophylaxis, and preemptive treatment of certain infections, including COVID‐19, could likely reduce morbidity and mortality.^10^ During the study period, up‐front immunoglobulin substitution in addition to prophylaxis for herpes and PJP gradually became a standard approach in Sweden, resulting in a 40% reduction in the infection rate.

In our study, patients with an extremely poor prognosis who were heavily pretreated were included in the compassionate‐use programs for teclistamab and elranatamab. The overall response rate (ORR) was 69% for BsAb treatment, and the median OS and PFS were not reached at 15 months, a finding that compares favorably with previously published data. We also conclude that hospitalization for full‐dose treatment might not be necessary. The immune effector cell encephalopathy (ICE) score may not be required for all MM patients treated with BsAbs, as the incidence of ICANS is negligible and of low grade. It appears that infections are the main risk associated with BsAb treatment, and appropriate prophylaxis should be initiated at the start of BsAb therapy and will at least result in a 50% risk reduction of the infection rate.

## AUTHOR CONTRIBUTIONS

**Katarina Uttervall:** Conceptualization (equal); data curation (equal); writing – original draft (supporting); writing – review and editing (equal). **Love Tätting:** Conceptualization (equal); formal analysis (lead); software (lead); writing – original draft (equal); writing – review and editing (equal). **Konstantinos Lemonakis:** Conceptualization (equal); validation (equal); writing – original draft (equal); writing – review and editing (supporting). **Mousa Majd:** Conceptualization (equal); writing – original draft (equal). **Jacob Crafoord:** Conceptualization (supporting); data curation (equal); visualization (equal); writing – original draft (equal); writing – review and editing (equal). **Mikael Olsson:** Conceptualization (equal); resources (equal); writing – original draft (supporting); writing – review and editing (equal). **Ulf‐Henrik Mellqvist:** Conceptualization (equal); writing – original draft (supporting). **Markus Hansson:** Conceptualization (equal); data curation (equal); resources (equal); writing – original draft (supporting). **Hareth Nahi:** Conceptualization (lead); formal analysis (lead); funding acquisition (lead); investigation (lead); software (equal); writing – original draft (lead); writing – review and editing (equal).

## FUNDING INFORMATION

This work was supported by [HN, cancerfonden, Dnr: 4–2528/2019]. The funders had no role in the study design, data collection and analysis, decision to publish, or preparation of the manuscript.

## CONFLICT OF INTEREST STATEMENT

KU: Consultancy for Janssen; advisory board for Janssen and Sanofi; lecture fees from BMS and Janssen. HN: Employee of Pfizer.

## ETHICS STATEMENT

The study was approved by the Institutional Review Board of (Dnr 2023–03123‐01). All procedures performed in studies involving human participants followed the ethical standards of the institutional and/or national research committee and with the 1964 Helsinki Declaration and its later amendments or comparable ethical standards.

## INFORMED CONSENT

Informed consent was obtained from all individual participants included in the study.

## Data Availability

The data that support the findings of this study are available on request from the corresponding author. The data are not publicly available due to privacy or ethical restrictions.
